# Menstrual migraines

**DOI:** 10.1186/1129-2377-16-S1-A23

**Published:** 2015-09-28

**Authors:** Franco Granella

**Affiliations:** Department of Neurosciences, University of Parma, Parma, Italy

The third edition of the International Classification of Headache Disorders (ICHD-3 beta), published in 2013, includes recommended criteria for “*A1.1.1 Pure menstrual migraine without aura*” (PMM) and “*A1.1.2 Menstrually related migraine without aura”* (MRM). The criteria are in the appendix while it is debated whether menstruation should be considered as a migraine trigger, or if menstrual migraine is a distinct clinical entity. Based on the ICHD-3 beta diagnostic criteria, menstrual attacks *must* occur on day 1 ± 2 (i.e., days -2 to +3) of menstruation in at least two out of three menstrual cycles, even though some studies have proposed a wider perimenstrual window. PMM and MRM may occur even in women taking combined oral contraceptives or hormone replacement therapy. In such cases, the mechanisms of migraine may be different, with endometrial bleeding resulting from the normal menstrual cycle and bleeding as a result of the withdrawal of exogenous hormones. When PMM or MRM are considered to be associated with exogenous oestrogen withdrawal, both codes A1.1.1 or A1.1.2 and *“8.3.3 Oestrogen withdrawal headache”* should be used. Menstrual attacks concern mostly migraine without aura (MO). However, cases of PMM and MRM with aura have been observed, both in clinic-based and population studies. In a recent population-based study carried out in Norway[1], menstrual migraine (MM) accounted for 22% of migraine among female migraineurs aged 30-34 years (5% of the general population), being in most cases MRM (18.6%). Besides MO, several MM with aura (2.7% of migraineurs) were observed and the addition to appendix of MM with aura was proposed. Several studies have found that, in women with MRM, menstrual attacks are longer, more severe, more disabling, and less responsive to symptomatic treatment[2]. In women from the general population, menstrual attacks would differ from nonmenstrual attacks only in women who fulfill the ICHD criteria for MM[3]. Other issues, as the relationship of MM to premenstrual syndrome, still await to be clarified.
